# Identification of diverse cell populations in skeletal muscles and biomarkers for intramuscular fat of chicken by single-cell RNA sequencing

**DOI:** 10.1186/s12864-020-07136-2

**Published:** 2020-10-31

**Authors:** Jinghui Li, Siyuan Xing, Guiping Zhao, Maiqing Zheng, Xinting Yang, Jiahong Sun, Jie Wen, Ranran Liu

**Affiliations:** 1 Institute of Animal Sciences, Chinese Academy of Agricultural Sciences; State Key Laboratory of Animal Nutrition, Key Laboratory of Animal (Poultry) Genetics Breeding and Reproduction, Ministry of Agriculture, Beijing, 100193 P. R. China; 2 Animal Breeding and Genomics, Wageningen University & Research, Wageningen, The Netherlands

**Keywords:** scRNA-seq, Breast muscle, Intramuscular fat, Cell cluster, RNA in situ hybridization

## Abstract

**Background:**

The development of skeletal muscle is closely related to the efficiency of meat production and meat quality. Chicken skeletal muscle development depends on myogenesis and adipogenesis and occurs in two phases—hyperplasia and hypertrophy. However, cell profiles corresponding to the two-phase muscle development have yet to be determined. Single-cell RNA-sequencing (scRNA-seq) can elucidate the cell subpopulations in tissue and capture the gene expression of individual cells, which can provide new insights into the myogenesis and intramuscular adipogenesis.

**Results:**

Ten cell clusters at the post-hatching developmental stage at Day 5 and seven cell clusters at the late developmental stage at Day 100 were identified in chicken breast muscles by scRNA-seq. Five myocyte-related clusters and two adipocyte clusters were identified at Day 5, and one myocyte cluster and one adipocyte cluster were identified at Day 100. The pattern of cell clustering varied between the two stages. The cell clusters showed clear boundaries at the terminal differentiation stage at Day 100; by contrast, cell differentiation was not complete at Day 5. *APOA1* and *COL1A1* were selected from up-regulated genes in the adipocyte cluster and found to be co-expressed with the *ADIPOQ* adipocyte marker gene in breast muscles by RNA in situ hybridization.

**Conclusions:**

This study is the first to describe the heterogeneity of chicken skeletal muscle at two developmental stages. The genes *APOA1* and *COL1A1* were identified as biomarkers for chicken intramuscular fat cells.

## Background

Chicken has become the largest consumer meat worldwide. The development of skeletal muscle closely relates to the efficiency of meat production and the quality of meat [1]. Chicken as a widely used developmental model significantly elucidates the molecular and cellular bases that control developmental processes.

In animals, skeletal muscle development depends on myogenesis and adipogenesis. Both myocytes and adipocytes originate from mesenchymal progenitor cells [2]. Their development occurs in two phases, the determination phase (hyperplasia) and the terminal differentiation phase (hypertrophy). Hyperplasia refers to the increase in the number of cells, which occurs mainly in the embryonic period as the numbers of adipocytes and muscle fibers are fixed by the day of birth or in early postnatal ages [3, 4]. Du et al. deduced that cattle muscle is in the hyperplasia phase from the middle stage of gestation to birth and then enters the hypertrophy phase [5]; chicken muscle was in the hyperplasia phase from the embryonic stage to Week 4 or Week 5. Seven weeks from birth, cells mainly increase in volume and enter the hypertrophy phase [4]. The skeletal muscle is a highly complex organ. However, the change in cell profiles corresponding to the two-phase muscle development has yet to be determined. Specifically, the deposition of intramuscular fat (IMF) can markedly promote the tenderness of meat and exerts an effect on the flavor of meat; however, the biomarker genes detected for IMF are quite limited.

Single-cell RNA sequencing (scRNA-seq) has significantly elucidated cell population diversity within tissues. This technique has provided insights into the heterogeneity of gene expression across cells, the trajectory of cell lineages during development, and the identification of cell-specific gene expression [6, 7]. Guo et al. conducted a systematic single-cell transcriptome analysis of more than 400,000 cells, covering all major mouse organs. The 17 cell subpopulations of leg muscles were defined [8]. A developmental hierarchy of adipose progenitors consisting of *DPP4*^+^ interstitial progenitors that generate committed *ICAM1*^+^ and *CD142*^+^ preadipocytes was defined by scRNA-seq, which are poised to differentiate into mature adipocytes [9].

In the current study, single-cell transcriptome sequencing by high-throughput scRNA-seq (10× Genomics Chromium) was conducted to clarify the diversity of the cell profiles of chicken breast muscles and identify marker genes for IMF. The heterogeneity of chicken breast muscles and the composition of cells, as well as the molecular characteristics of muscles and IMF cells, were analyzed at two distinct developmental stages. Typical cell-specific expressed genes were verified by RNA in situ hybridization [10–12].

## Results

### Single-cell transcriptome profiling at two developmental stages

The breast muscles at Day 5 (D5) and Day 100 (D100) were used to represent the two developmental stages of the skeletal muscle—hyperplasia and hypertrophy. In total, single-cell transcriptomes of 8948 cells at D5 and 4504 at D100 were obtained (Table S1). At D5, 13,725 genes were detected, with an average of 826 unique molecular identifiers (UMIs) and 264 genes expressed per cell. At D100, 10,917 genes were detected, with an average of 218 UMIs and 107 genes expressed per cell. The mean reads of the cells at D5 and D100 were 43.399 k and 94.599 k, respectively. Samples of the two stages were subjected to sequencing saturation (Fig. S1). There were 4225 poor-quality cells at D5 and 2679 at D100 were removed based on filtration criteria used, including the proportion of mitochondrial genes expressed and the number of expressed gene per cell (Table S2). The remain 4723 cells at D5 and 1825 cells at D100 were used for subsequent analysis. At D5, the mean UMI count in each cluster ranged from 609 to 1708, and the mean UMI count of all filtrated cells was 1055. At D100, the mean UMI count of each cluster ranged from 309 to 641, and the mean UMI count of all cells was 435. The number of expressed genes in each cluster ranged from 265 to 455 at D5 and 154 to 397 at D100 (Table S3).

### Differences in the cell type of breast muscle at day 5

**Fig. 1:**
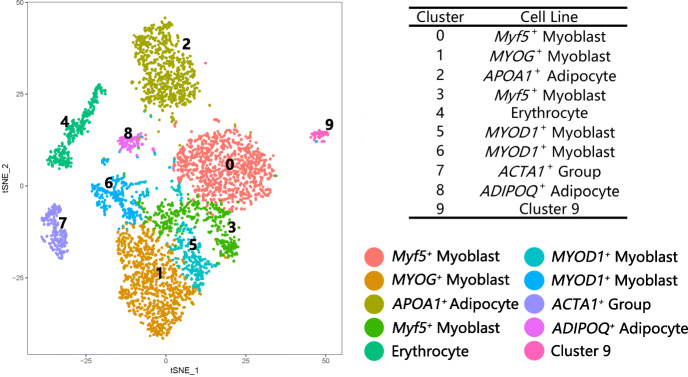
The t-SNE clustered results for the cells at D5. Clusters 0, 1, 3, 5, and 6 were identified as myoblasts; Clusters 2 and 8 were identified as adipocyte clusters; Cluster 4 was an erythrocyte cluster; Cluster 7 was an *ACTA1*^+^ cluster; and Cluster 9 was undetermined owing to the lack of expression of a known marker gene

At D5, 4723 cells were clustered based on gene expression similarity, and 10 cell populations were identified (Fig. 1). The cell populations with their corresponding proportions were as follows: Cluster 0, 22.6%; Cluster 1, 20.5%; Cluster 2, 17.3%; Cluster 3, 9%; Cluster 4, 7.7%; Cluster 5, 7%; Cluster 6, 6.5%; Cluster 7, 5.3%; Cluster 8, 2.5%; and Cluster 9, 1.6%. The top 20 up-regulated genes in each cluster were used to construct the heatmap (Fig. S2).

**Fig. 2:**
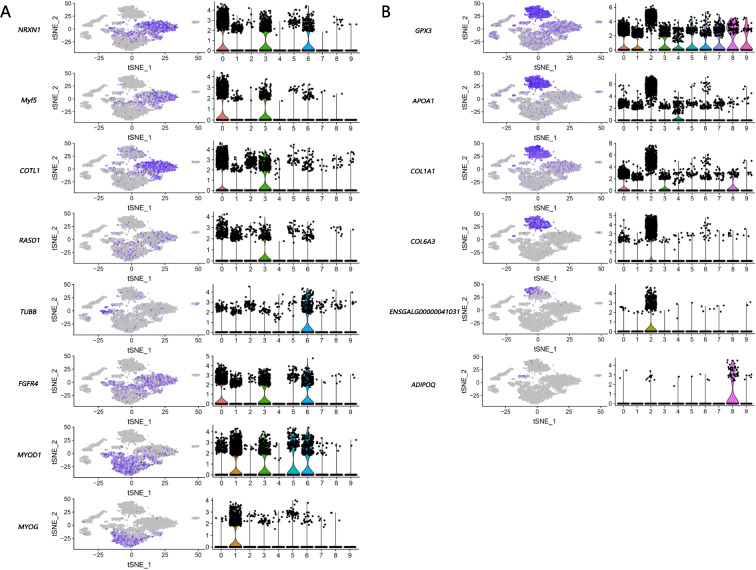
Individual gene t-SNE and violin plots showing the expression levels and distribution of representative marker genes at D5. Fig. **a** presents the details of the eight functional genes of muscle cells (*NRXN1*, *Myf5*, *COTL1*, *RASD1*, *TUBB*, *FGFR4*, *MYOD1*, *MYOG*) and Fig. **b** presents the six functional genes of adipocytes (*GPX3*, *APOA1*, *COL1A1*, *COL6A3*, *ENSGALG00000041031*, *ADIPOQ*). The figures on the left are the heatmap of genes, where the expression of the genes in all cells can be visualized, and the expression level gradually increases from gray to purple. The figures on the right are the violin plots for a given gene. The abscissa represents the cell clusters, and the ordinate represents the normalized read count in log scale for the genes

Clusters 0, 1, 3, 5, and 6 were identified as myoblasts, which up regulated *Myf5*, *MYOD1*, *MYOG*, and other marker genes compared to other clusters. Known functional genes—*NRXN1*, *COTL1*, *RASD1*, *TUBB*, and *FGFR4*—were also found to be up-regulated in these five myoblast subpopulations (Fig. 2a).

Cluster 8 was identified as an adipocyte population on the basis of the high expression of the marker genes—for instance, *ADIPOQ* and *FABP5*. *ADIPOQ* expressed in adipose tissue reportedly acts as a marker gene for mature adipocytes. *FABP5* participates in the peroxisome proliferator activated receptor (PPAR) signaling pathway; in addition, *FABP5* transports and binds to fatty acids and may play a role in fatty acid uptake, transport, and metabolism [13]. The remaining subpopulations without expressed known marker were characterized by pathway enrichment analysis of genes. Cluster 2 was described as another adipocyte population because the up-regulated genes were enriched in pathways related to fat deposition. The PPAR signaling pathway, Wingless-related integrated site (Wnt) signaling pathway, and extracellular matrix (ECM)–receptor interaction, among others, were associated with fat deposition signaling pathways [14–17]. *GPX3*, *APOA1*, *COL1A1*, *COL6A3*, and *ENSGALG00000041031*, among others, were up regulated in Cluster 2, and *ADIPOQ* was up regulated in Cluster 8 (Fig. 2b).

Enriched biological processes and molecular functions were related to iron ion binding and oxygen transport in Cluster 4, which was identified as the erythrocyte population. Cluster 7 was identified as *ACTA1* group with high expression of *ACTA1,* which was one of marker genes for human stem cells [18]. Cluster 9 was uncertain because of the lack of a known marker for gene expression or a known enriched pathway.

### Differences in the cell type of breast muscle at day 100

**Fig. 3:**
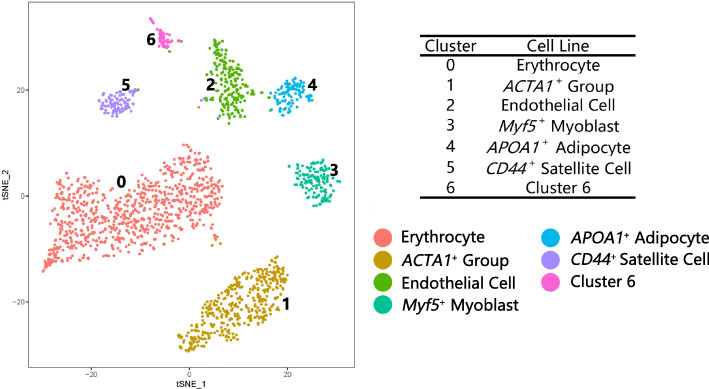
The t-SNE clustered results of the cells at D100. Cluster 0 was defined as an erythrocyte cluster; Cluster 2 was an endothelial cell cluster; Cluster 3 was a myoblast cluster expressing *Myf5*; Cluster 4 was identified as an adipocyte cluster; Cluster 5 consisted of satellite cells; Cluster 1 was the *ACTA1*^+^ cluster; and cluster 6 was undetermined owing to the lack of expression of a known marker gene

A total of 1825 cells at D100 were assigned to seven cell clusters (Fig. 3) with the following proportions: Cluster 0, 45%; Cluster 1, 22%; Cluster 2, 11%; Cluster 3, 7%; Cluster 4, 6%; Cluster 5, 6%; and Cluster 6, 3%. The top 20 up-regulated genes in each cluster were used to construct the heatmap (Fig. S3). The cell populations at D100 showed clear boundaries.

**Fig. 4:**
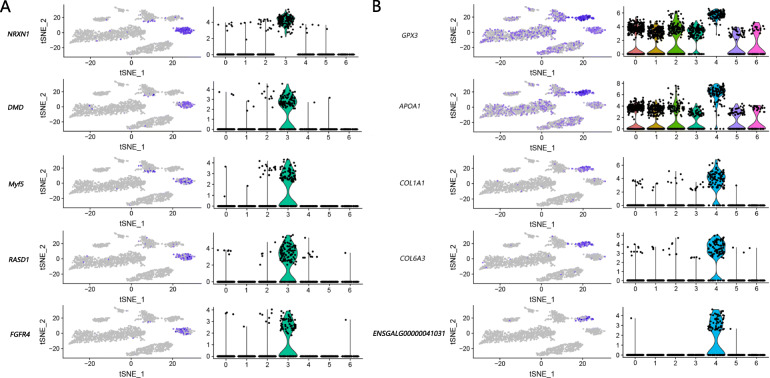
Individual gene t-SNE and violin plots showing the expression levels and distribution of representative marker genes at D100. Fig. **a** shows the details of five functional genes of muscle cells (*NRXN1*, *DMD*, *Myf5*, *RASD1*, *FGFR4*). Fig. **b** shows five genes of adipocytes (*GPX3*, *APOA1*, *COL1A1*, *COL6A3*, *ENSGALG00000041031*). The figures on the left are the gene heatmap, and the gene expression in all cells can be visualized. The expression level gradually increases from gray to purple. The figures on the right are the violin plot for the genes. The abscissa represents the cell clusters, and the ordinate represents the gene expression level

Cluster 0 at D100 was defined as the erythrocyte population. The GO enrichment results were related to iron ion binding and oxygen transport. On the basis of the characteristics of *TMSB4X*, *GNG11*, and *RHOA* expression in Cluster 2, the population was identified as an endothelial cell population. Cluster 3 was defined as the myoblast population because the expression of the marker gene *Myf5* was up-regulated. The expression of *NRXN1*, *DMD*, *RASD1*, *FGFR4*, and other genes were also up-regulated (Fig. 4a). Cluster 4 was identified as the adipocyte population in which all up-regulated genes were enriched in the Wnt signaling pathway, ECM–receptor interaction, TGF-beta signaling pathway, and so on. *APOA1*, *COL1A1*, *GPX3*, *COL6A3*, *ENSGALG00000041031*, and other genes were up regulated (Fig. 4b). The genes *CD29*, *CD44*, and *CXCR4* were up-regulated in Cluster 5, and it was defined as satellite cells. Cluster 1 was identified as *ACTA1* group with high expression of *ACTA1,* which was one of marker genes for human stem cells [18]. Cluster 6 was undefined owing to the absence of any known marker gene expressed.

For population assignments at D5 and D100, some clusters were similar between two stages on the basis of the expression of typical genes and their enriched pathways, including myocyte and adipocyte clusters.

### Commonly expressed genes at two stages in myoblast and adipocyte populations

A total of 3097 cells were categorized into five clusters (Clusters 0, 1, 3, 5, and 6) related to the myocyte at D5, and 133 cells were assigned to one myocyte cluster (Cluster 3) at D100. Meanwhile, 433 genes expressed in myocyte clusters were expressed at D5, and 186 genes were expressed at D100, 111 of which were commonly expressed at two stages. Among the commonly expressed genes, 28 were up-regulated, whereas 83 were down-regulated at D100. Moreover, 20 genes, including *RASD1*, *NRXN1*, *S100A1*, *SPTBN1*, *Myf5*, and *COTL1*, were functional genes involved in muscle formation and development (Table S4).

**Fig. 5:**
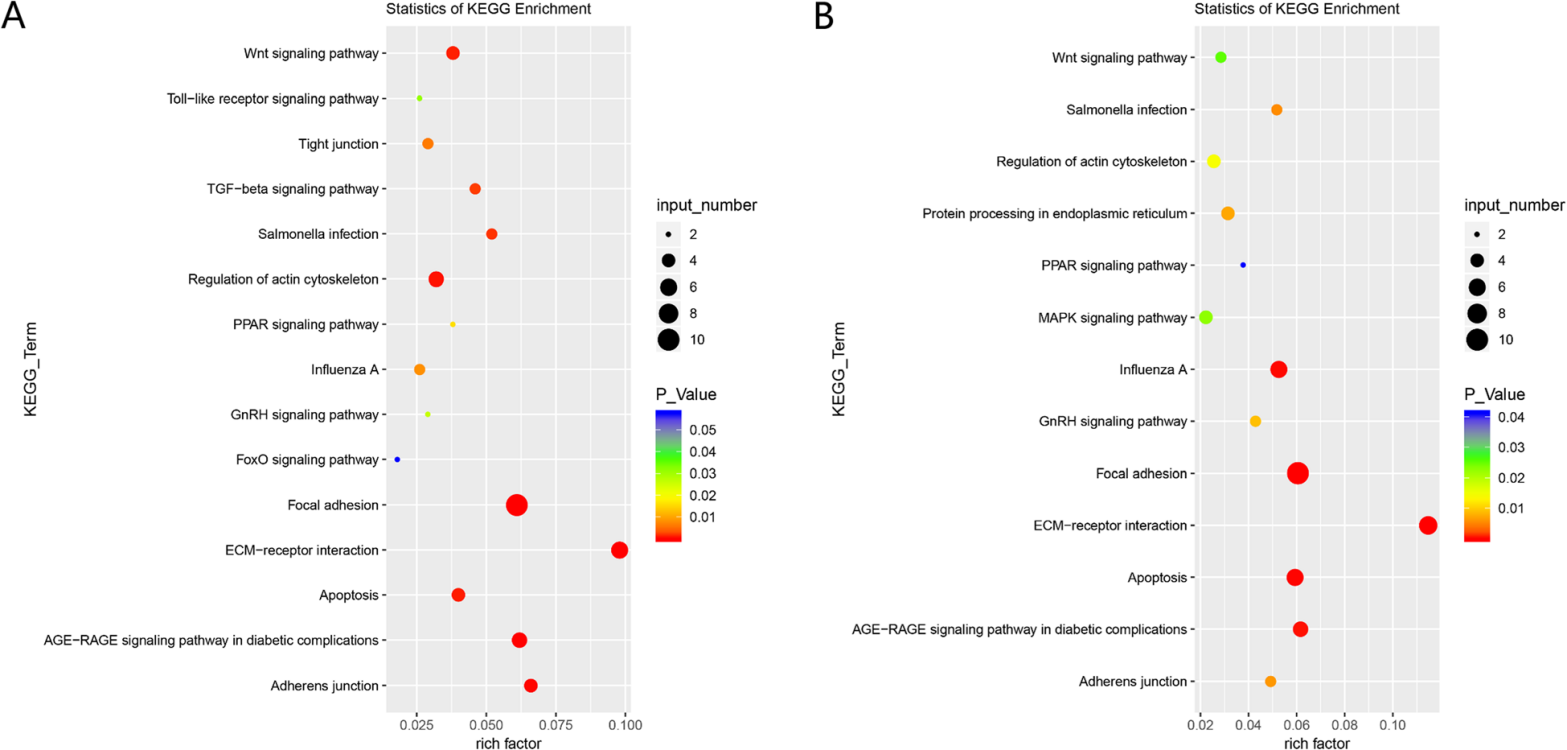
The KEGG pathway enrichment of the up-regulated genes intramuscular adipocyte clusters at D5 (**a**) and D100 (**b**)

A total of 935 cells were categorized into two clusters (Clusters 2 and 8) related to adipocytes (IMF) at D5; 106 cells were assigned to one adipocyte cluster (Cluster 4) at D100. Meanwhile, 143 genes in the adipocyte cluster were expressed at D5, and 114 genes were expressed at D100; 63 genes were commonly expressed at two stages. Both adipocyte populations at D5 and D100 were significantly enriched in the Wnt signaling pathway, PPAR signaling pathway, focal adhesion, ECM–receptor interaction, apoptosis, and advanced glycation end products–receptor for advanced glycation end products (AGE-RAGE) signaling pathway in diabetic complications (Fig. 5). Of the 63 genes, 29 were up-regulated, and 34 were down-regulated. In addition, 19 genes were functional genes associated with the development and lipid transport of adipocytes. These genes included *KLF2*, *GPX3*, *JUN*, *ENSGALG00000041031*, *APOA1*, *S100A10*, *MMP2*, *SERPINF1*, and *KLF6* (Table S5).

### Verification of marker gene expression in breast muscles with RNA in situ hybridization

**Fig. 6:**
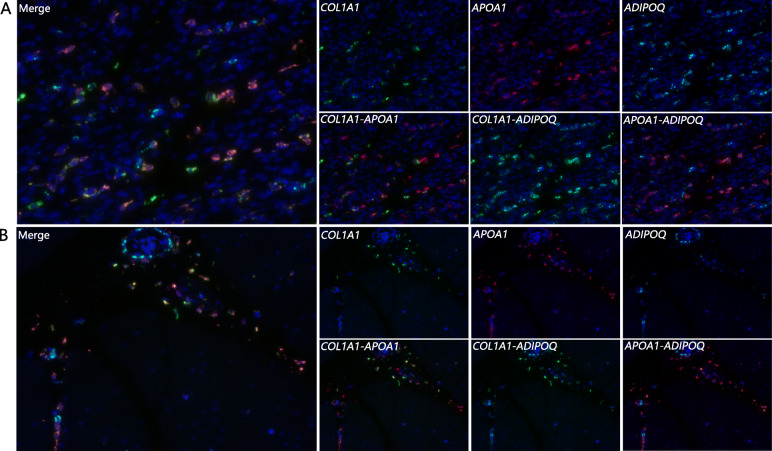
In situ validation of the mRNA expression of the adipocyte marker *APOA1*, *COL1A1*, and *ADIPOQ* in breast muscle. *APOA1* and *COL1A1* are selected from the up-regulated genes in intramuscular adipocytes. *ADIPOQ* is a known adipocyte marker gene and used as a positive control. Fig. **a** shows the results in the breast muscle of Jingxing-Huang chicken H lines at D5. Fig. **b** presents the results of those at D66. The co-stained fluorescence images of the three genes are merged. The first row from left to right is a single-channel image of each gene (*n* = 5). *COL1A1* exhibits green fluorescence; *APOA1,* red fluorescence; and *ADIPOQ,* blue fluorescence. The blue background is DAPI. The images on the second row from left to right represent the dual-channel merged images of *COL1A1* and *APOA1*, *COL1A1* and *ADIPOQ*, and *APOA1* and *ADIPOQ*

The biomarker genes for intramuscular adipocytes were given focus in the current study. On the basis of the aforementioned scRNA-seq results, two up-regulated genes in the intramuscular adipocyte cluster—*APOA1* and *COL1A1—*were selected and verified by RNA in situ hybridization in breast muscles. The known adipocyte marker gene *ADIPOQ* [9] was tested as positive control. As shown in Fig. 6, *APOA1*, *COL1A1*, and *ADIPOQ* are co-expressed in the breast muscle. The patterns of expression of *APOA1* and *COL1A1* in the breast muscle were similar to that of *ADIPOQ* at two different developmental stages.

## Discussion

Skeletal muscles comprise the largest proportion of meat produced by animals. The general development of skeletal muscles can be divided into two phases— hyperplasia and hypertrophy. In this study, two phases of muscle development were examined and characterized by high-throughput scRNA-seq. The dataset of the reported single-cell transcriptomes is thus far the first to describe heterogeneity in chicken breast muscle cells in two developmental stages, although a large number of studies have indicated heterogeneity in tumors or stem cells [19–22].

Based on deep sequencing and analysis of single-cell transcriptomes of breast muscle tissues in two developmental stages, the heterogeneous cell population in the breast muscle was identified by the known cell marker genes and/or functional genes of different cell populations. Five clusters (0, 1, 3, 5, and 6) at D5 and one cluster (3) at D100 were identified as myoblasts, based on the up-regulated genes—*Myf5, MYOG*, *or MYOD1*. *Myf5* was an early differentiation-specific gene expression of myoblasts; *MYOD1* and *MYOG* were expressed during myoblast differentiation; and *MYOG* was expressed during late-stage myoblast differentiation [3, 23]. *Pax3*-induced myogenic progenitor cells express not only the transcription factors *Pax3* and *Myf5* but also the cell surface markers *CD29*, *CD44*, *Calpain-2*, and *CXCR4* [24]. Cluster 5 in the sample at D100 up regulated *CD29*, *CD44*, and *CXCR4* and was identified as a myogenic satellite population. *ACTA1* was expressed in the early differentiation stage of myogenic satellite cells [18] and could be detected in chicken breast muscle in the embryonic stage (ED12–ED17) [25]. In the current study, *ACTA1* expression was detected in Cluster 7 (5.3%) at D5 and Cluster 2 (22%) at D100. In these clusters, the mean expression levels of *ACTA1* were higher (37.95–59.72) than the general mean expression levels (1.29–2.25). *ACTA1* is one of marker genes for human stem cells [18]. However, more evidence is need to determine whether the *ACTA1* groups defined potentially consisted of stem cells or not. The abnormal proportion of the *ACTA1* group at D100 might be attributable to cell damage and the removal of a large proportion of poor-quality cells. Specifically, cells with a large proportion of mitochondrial gene expression could be caused by cell damage. Skeletal muscle cells with more than 15% or 20% of mitochondrial gene expression were removed as low-quality cells [26, 27]. In the current study, a percentage of mitochondrial gene expression in cells at D100 had peaks around 20 and 60%. The 60% peak was obviously high and suggested the occurrence of cell damage. Thus, the proportion of different cell types was abnormal in cells at D100, including the large proportion of erythrocytes and the *ACTA1* group showed. The quantity and proportion of different cell types at the late developmental stage need to be subsequently investigated.

Myo–adipogenic balance in skeletal muscles is important for both human skeletal muscle dysfunction and myopathies, as well as animal meat quality. As the minority cells in muscles, the adipocyte-related population is defined by a combination of expressed functional genes. The PPAR and Wnt signaling pathways are associated with both adipocyte development and IMF deposition [14, 28, 29]. In the currently defined adipocyte population (Clusters 2 and 8 in D5, Cluster 4 in D100), *SERPINF1*, *RHOA*, *JUN*, and *TCF7L2* were enriched in the Wnt signaling pathway; moreover, *APOA1* and *ENSGALG00000004509*, among others, were enriched in the PPAR signaling pathway (Tables S6, S7).

Notably, the status of the clustered cells showed distinct patterns between the two developmental stages tested in which transitional cell clusters existed and myoblast differentiation was not complete at D5. By contrast, the boundaries of the cell clusters, which were at terminal differentiation stages, appeared rather clear at D100.

The integrative t-SNE analysis of two samples at D5 and D100 were applied and the results showed 12 clusters (Fig. S4). Merged data generated seven similar clusters as stated above and includes two adipocyte clusters, four myoblast clusters, erythrocyte cluster, *ACTA1* Group and *CD44* satellite cluster. There is no new cluster could be identified because of lacking a known marker for gene expression or a known enriched pathway.

When commonly expressed genes related to the myocyte and adipocyte populations at different developmental stages were explored, 111 genes related to the myocyte population were found. The majority of differentially expressed genes (83/111) were down-regulated. There were 63 genes from the adipocyte population were commonly expressed in the two stages, and the up-regulated and down-regulated genes were almost half and half. The commonly expressed genes showed potential as marker genes for different cell types. Of the aforementioned commonly expressed genes, 20 genes were known functional genes for muscle development or growth, and 19 genes were known functional genes for fat development (Tables S4, S5).

A major advantage of high-throughput scRNA-seq is the identification of cell type-specific expressed genes. The level of IMF is an important index in animal breeding, and a molecular marker for both breeding technology and research is desired. Giordani et al. [26] showed that several adipocytes were detected by single-cell sequencing of primary cells isolated from skeletal muscle. The adipocytes only comprised 0.502% (39/7775) of the total cells captured. To identify the biomarker for intramuscular adipocytes, a higher adipocyte capture efficiency was preferred in the current study. Thus, two single-cell suspensions containing muscle cells and adipocytes were mixed in a ratio of 1:3. In our results, the proportions of primary adipocytes were 19.8 and 6% in the two stages. The adipocyte capture efficiency improved using our strategy, although the proportion of each sub-cell type did not represent the actual composition of the tissue.

RNA in situ hybridization has been widely used to verify newly characterized genes from scRNA-seq studies. Two up-regulated genes— the apolipoprotein A1 encoding gene (APOA1) and the type I collagen encoding gene (COL1A1) —in the intramuscular adipocyte population and one known mature adipocyte marker gene *ADIPOQ* were verified by RNA in situ hybridization in two developmental stages. *APOA1* was identified as a candidate for IMF deposition according to the proteomics of breast muscle in local breeds in our previous study [25]. In the current study, the locations of *APOA1* and *COL1A1* expression in breast muscle were tested and found to be consistent with the positive control *ADIPOQ*. *APOA1* performed better than *COL1A1* in signal distribution and intensity. Collectively, *APOA1* and *COL1A1* were identified for the first time as marker genes for intramuscular adipocytes as confirmed by scRNA-seq and RNA in situ hybridization.

## Conclusions

Our study is the first to describe the heterogeneity in the chicken skeletal muscle. Cell-specific expressed genes for myocytes and adipocytes in skeletal muscle were supplied. The results suggested that the cell differentiation was not complete at the early developmental stages after hatching. *APOA1* and *COL1A1* can be used as marker genes for intramuscular fat study.

## Methods

### Ethics statement

All experimental procedures with chickens were performed according to the Guidelines for Experimental Animals established by the Ministry of Science and Technology (Beijing, China). Ethical approval on animal survival was given by the Animal Ethics Committee of the Institute of Animal Sciences (IAS), Chinese Academy of Agricultural Sciences (CAAS, Beijing, China) with the following reference number: IASCAAS-AE-03.

### Experimental animals

An inbred dwarf Jingxing–Huang chicken line with a relatively high capacity to deposit IMF was used in the current study [30]. Chickens aged 5 d (*n* = 4) and 100 d (*n* = 1) were obtained from the Institute of Animal Sciences, Chinese Academy of Agricultural Sciences (Beijing, China) and used to represent the hyperplasia and hypertrophy stages [4]. Tissue samples from the Jingxing–Huang chicken H line were collected from the Institute of Animal Sciences, Chinese Academy of Agricultural Sciences at D5 (*n* = 5) and D66 (n = 5) and used for RNA in situ hybridization. The body weights of the H line at D5 and D66 were similar to those of the dwarf Jingxing–Huang chickens at D5 and D100, respectively. All animals were fed ad libitum, and standardized feeding management was conducted. Animal euthanasia was approved by the Animal Ethics Committee of the Institute of Animal Sciences in Beijing, China.

### Preparation of the single-cell suspension

Chickens were stunned by electrical stunning (120 mA, 50 Hz) and then slaughtered with a quick, exsanguination by severing the carotid artery. After being sprayed with 75% ethanol, the pectoralis major muscle was isolated, removing 10 g of the muscle from which primary cells were extracted. The fresh muscles were washed 3 times in phosphate-buffered saline (PBS) (Hyclone, Logan, Utah, USA). The fascia and blood were removed. The muscle was minced into 1 mm^3^ segments and then digested with 0.1% w/v type I collagenase (Gibco, Life Technologies, Foster City, CA, USA). For the tissue mixed with a digestive liquid, the volume ratios used were 1:10 at D5 and 1:5 at D100. The tube was heated to 37 °C and shaken at 70 r/min and then digested in an air bath. Cells from the D5 chicken were digested for 80 min, and cells from the D100 chickens were digested for 40 min. After digestion, the viscous mixture was combined with an equal volume of the complete medium consisting of Dulbecco’s modified Eagle’s medium (DMEM)/F12 (Gibco, Life Technologies, Foster City, CA, USA), 10% fetal bovine serum (Gibco, Life Technologies, Foster City, CA, USA), and 1% penicillin/streptomycin (Gibco, Life Technologies, Foster City, CA, USA). The mixture was centrifuged at 1500 r/min for 10 mins to obtain a liquid in the upper layer (with mature fat content) and a precipitate in the bottom layer. Up to 2 mL of the upper liquid oil containing IMF cells were absorbed carefully and then centrifuged at 1500 r/min for 10 mins to filter impurities. The remaining liquid and precipitate were passed through 100-, 200-, 400-, and double 600-mesh cells. The sieve was filtered, and the resulting liquid was centrifuged at 1500 r/min for 10 min. The resulting precipitate consisted of other cells containing muscle cells. These cells were then suspended in 2 mL of the PBS solution.

### Single-cell sequencing with 10× genomics

The extracted primary muscle cells and adipocytes were sequentially subjected to preliminary quality inspection. Cell concentration and viability were determined using a Countstar BioMed Professional Immune Cell Counter (Life Science, Shanghai, China). Two single-cell suspensions of muscles containing cells and adipocytes were mixed. The volumes of liquid with similar concentrations were mixed in a ratio of 1:3 for the liquid and upper-layer cells. The mixed cell suspension was then adjusted to the ideal concentration of 1000/μL. The Chromium Single Cell Controller (10× Genomics, San Francisco, CA) was used to analyze 3000 recovered cells. The RNA from the barcoded cells was subsequently reverse-transcribed, and sequencing libraries were constructed with reagents from a Chromium Single Cell 3′ v2 Reagent Kit (10× Genomics) in accordance with the instructions provided by the manufacturer. Sequencing was performed with Illumina HiSeq 4000 as instructed by the manufacturer (CapitalBio Technology, Beijing, China). The sequencing length consisted of two parts: (i) 26 bp Read1, including a 16 bp barcode and a 10 bp UMI, and (ii) 98 bp Read2, which was the sample RNA sequence.

### Sequencing data quality assessment and data processing

Regular data analysis was mainly performed using CapitalBio Technology (Beijing, China). To ensure the data availability and validity of subsequent analysis, the sequencing error rate was first evaluated. The percentage of Q30 in the barcode, RNA read, and UMI in the total base was detected. The STAR software [31] was used to align Read2 with the reference genome [Ensembl Gallus_gallus-5.0 (ftp://ftp.ensembl.org/pub/release-92/fasta/gallus_gallus/dna/Gallus_gallus.Gallus_gallus-5.0.dna.toplevel.fa.gz)] with the Cell Ranger version 2.0 by using default parameters (10× Genomics, Pleasanton, CA). The Maximal Mappable Prefix (MMP) from the first base of the read was determined, and the unique aligned sequence and UMI sequences were selected [32], and the UMI was corrected to remove duplicate PCR products [33].

Downstream single-cell analyses were performed using the R package Seurat version 2.0 with default parameters unless otherwise stated [34]. The D5 and D100 datasets were analyzed independently. The high expression of mitochondrial genes in cells could be indicators of poor sample quality or muscle cells with increased mitochondrial gene expression, affecting the classification of the cell population [35]. In the present study, the cells were removed if their proportions of mitochondrial gene expression were > 30% at D5 and > 25% at D100 (Fig. S5, Table S2).

### Dimensionality reduction, clustering, and differential gene expression analysis

Dimensionality reduction, clustering, and differential gene expression analysis were conducted using Seurat version 2.0 with default parameters unless otherwise stated. PCA was applied, and the first 10 and 7 principal components generated from the D5 and D100 datasets were used for clustering and visualization (Fig. S6). A two-dimensional map of the cell populations was generated using the t-distributed stochastic neighbor embedding (t-SNE). Clusters were identified using the Seurat FindClusters function with a resolution parameter of 0.6 for the two stages. The up-regulated gene or differentially expressed gene was defined as the differentially expressed genes in each cluster relative to all other clusters. Genes with *P* < 0.01 and |LogFC> 0.25|were differentially expressed genes of each cluster. The top 20 up-regulated genes in each cluster, with *P* < 0.01 and LogFC> 0.25, were selected to construct the heatmap and violin plot.

### KEGG and gene ontology analysis

All characteristic genes expressed in each cell subpopulation were subjected to KEGG enrichment and GO enrichment by KOBAS 3.0 [36] to analyze the function of each cell subgroup and screening of genes related to the IMF.

### Verifying gene expression by RNA in situ hybridization

Three typical up-regulated genes in the adipocyte cluster were selected for RNA in situ hybridization to verify their expression [37] within the tissue at D5 and D66. Fresh tissue was immediately placed in a fresh 10% neutral formalin tissue fixative, fixed at room temperature for 32 h, embedded in paraffin, and sectioned. The paraffin tissue sections measuring 5 ± 1 mm were used for RNA in situ hybridization. Probes for 3 genes—*APOA1*, *ADIPOQ*, *COL1A1*—were designed by Advanced Cell Diagnostics. Subsequently, mRNA transcription was visualized using the RNAscope Multiplex Fluorescent Reagent v2 kit (Advanced Cell Diagnostics, San Francisco, California, USA) in accordance with the instructions provided by the manufacturer. For each experiment, *ADIPOQ* was used as positive control. Muscle sections were pretreated with a hydrogen peroxide solution, a target retrieval solution, and protease. The pretreated sections were ultimately hybridized with the RNA probe of the target gene for 2 h at 40 °C in a hybrid furnace, followed by a series of signal amplifications. After RNA in situ hybridization, the nuclei were counterstained with DAPI (4′,6-diamidino-2-phenylindole, a dye that can stain the nucleus in blue) for 30 s at room temperature. Images were obtained using the Vectra 3.0 Quantitative pathological imaging system (PerkinElmer, USA). Three probes were located in different probe channels, and the RNAscope probes of the 520, 570, and 620 channels were labeled with different fluorescence tags by using the TSA Plus fluorophore (Advanced Cell Diagnostics, San Francisco, California, USA).

## Supplementary information

**Additional file 1: Figure S1.** Sequencing saturation and median genes per cell at D5 and D100. Figs. A and C show sequencing saturation curves at D5 and D100, respectively. Sequencing saturation approaches 1.0 (100%) when all converted mRNA transcripts are sequenced. The dotted line is drawn at a value reasonably approximating the saturation point. Figs. B and D are the median genes per cell at D5 and D100, respectively.

**Additional file 2: Figure S2.** Heatmap of the top 20 up-regulated genes in each cluster at D5. The abscissa represents the cell clusters, and the ordinate represents the up-regulated genes in each cluster. The color changes from purple to yellow, indicating the gradual increase in gene expression.

**Additional file 3: Figure S3.** Heatmap of the top 20 up-regulated genes in each cluster at D100. The abscissa represents the cell clusters, and the ordinate represents the up-regulated genes in each cluster. The color changes from purple to yellow, indicating the gradual increase in gene expression.

**Additional file 4: Figure S4.** The t-SNE analysis of the data of two samples. Fig. A was the t-SNE result integrative results. Fig. B was the t-SNE results of D5 and D100, respectively. They merged to generate Fig. A.

**Additional file 5: Figure S5.** Distribution of data for quality assessment and data processing. A and B present the violin plots for the number of genes, UMI, and proportions of mitochondrial gene expression in detected cells at D5 and D100, respectively. C and D are the scatter plots of gene dispersion at D5 and D100, respectively. The ordinate represents the dispersion of the gene expression.

**Additional file 6: Figure S6.** Principal component standard deviation scatter plot at D5 (Fig. A) and D100 (Fig. B). The abscissa represents the principal component, and the ordinate represents the standard deviation of different principal components.

**Additional file 7: Table S1.** Cell counts at D5 and D100. **Table S2.** Cell filtration criteria at D5 and D100. **Table S3.** Mean unique molecular identifiers and mean genes in each cluster at D5 and D100. **Table S4.** Common differentially expressed genes of myoblast populations between the two developmental stages. **Table S5.** Common differentially expressed genes of adipocyte population between the two developmental stages. **Table S6.** KEGG enrichment pathways and related genes in Cluster 2 at D5. **Table S7.** KEGG enrichment pathways and related genes in Cluster 4 at D100.

## Data Availability

The single-cell RNA sequencing clean data reported in this paper have been deposited in the Genome Sequence Archive [38] in BIG Data Center [39] under accession number CRA002353, which can be publicly accessed at https://bigd.big.ac.cn/gsa/ . The reference genome (Gallus_gallus-5.0) data used in this study is available at ftp://ftp.ensembl.org/pub/release-92/fasta/gallus_gallus/dna/Gallus_gallus.Gallus_gallus-5.0.dna.toplevel.fa.gz .

## Abbreviations

ACTA1: Actin Alpha 1
ACTG1: Actin Gamma 1
ADIPOQ: Adiponectin
AEBP1: AE Binding Protein 1
AGE-RAGE: Advanced Glycation Endproducts–receptor for Advanced Glycation Endproducts
APOA1: Apolipoprotein A1
CD29: Integrin Subunit Beta 1
CD44: CD44 Molecule
CD81: CD81 Molecule
CNN3: Calponin 3
COL3A1: Collagen Type III Alpha 1 Chain
COL5A2: Collagen Type V Alpha 2 Chain
COL6A3: Collagen Type VI Alpha 3 Chain
COTL1: Coactosin Like F-Actin Binding Protein 1
CXCR4: C-X-C Motif Chemokine Receptor 4
D100: Day 100
D5: Day 5
DAPI: 4′,6-diamidino-2-phenylindole
DDX5: DEAD-Box Helicase 5
DMD: Dystrophin
DMEM: Dulbecco’s Modified Eagle’s medium
DPP4: Dipeptidyl Peptidase 4
ECM: Extracellular Matrix
ED: Embryonic Day
EEF1A1: Eukaryotic Translation Elongation Factor 1 Alpha 1
FABP5: Fatty Acid Binding Protein 5
FGFR4: Fibroblast Growth Factor Receptor 4
GNG11: G Protein Subunit Gamma 11
GPX3: Glutathione Peroxidase 3
HNRNPDL: Heterogeneous Nuclear Ribonucleoprotein D Like
HSP90AA1: Heat Shock Protein 90 Alpha Family Class A Member 1
ICAM1: Intercellular Adhesion Molecule 1
IMF: intramuscular fat
JUN: Transcription factor AP-1
KLF2: Kruppel Like Factor 2
KLF6: Kruppel Like Factor 6
KNN: K-nearest neighbor
LUM: Lumican
MMP: Maximal Mappable Prefix
MMP2: Matrix Metallopeptidase 2
Myf5: Myogenic factor 5
MYOD1: Myogenic Differentiation 1
MyoG: Myogenin
NRXN1: Neurexin 1
PAX3: Paired Box 3
PBS: Phosphate Buffer Saline
PCA: Principal Component Analysis
PPAR: Peroxisome Proliferator Activated Receptor
PPIB: Peptidylprolyl Isomerase B
PTGDS: Prostaglandin D2 Synthase
RASD1: Ras Related Dexamethasone Induced 1
RHOA: Ras Homolog Family Member A
S100A1: S100 Calcium Binding Protein A1
S100A10: S100 Calcium Binding Protein A10
scRNA-seq: Single-cell RNA Sequencing
SERPINF1: Serpin Family F Member 1
SPARC: Secreted Protein Acidic And Cysteine Rich
SPTBN1: Spectrin Beta, Non-Erythrocytic 1
TCF12: Transcription Factor 12
TCF7L2: Transcription Factor 7 Like 2
TGF-beta: Transforming Growth Factor Beta
TMSB4X: Thymosin Beta 4 X-Linked
t-SNE: t-distributed Stochastic Neighbor Embedding
TUBB: Tubulin Beta Class I
UMI: Unique Molecular Identifier
VIM: Vimentin
Wnt: Wingless-related integrated site
