# Supplementary figures and images for: Identification of diverse cell populations in skeletal muscles and biomarkers for intramuscular fat of chicken by single-cell RNA sequencing
Source: BMC Genomics. 2020 Oct 31;21:752. doi: 10.1186/s12864-020-07136-2 (PMC7603756; doi:10.1186/s12864-020-07136-2)

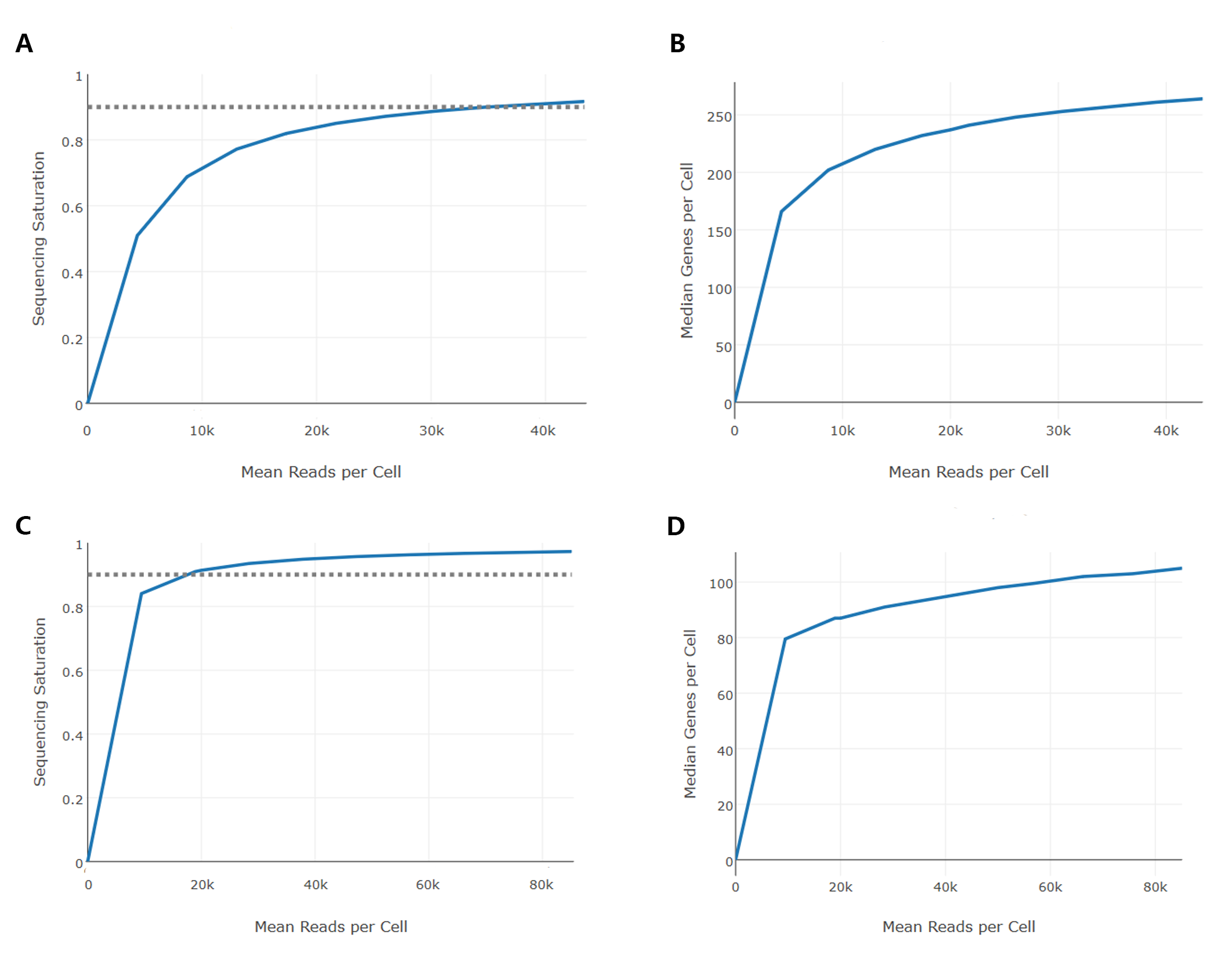

Supplement: Supplementary file 1 — Additional file 1: Figure S1. Sequencing saturation and median genes per cell at D5 and D100. Figs. A and C show sequencing saturation curves at D5 and D100, respectively. Sequencing saturation approaches 1.0 (100%) when all converted mRNA transcripts are sequenced. The dotted line is drawn at a value reasonably approximating the saturation point. Figs. B and D are the median genes per cell at D5 and D100, respectively. [file 12864_2020_7136_MOESM1_ESM.tif]

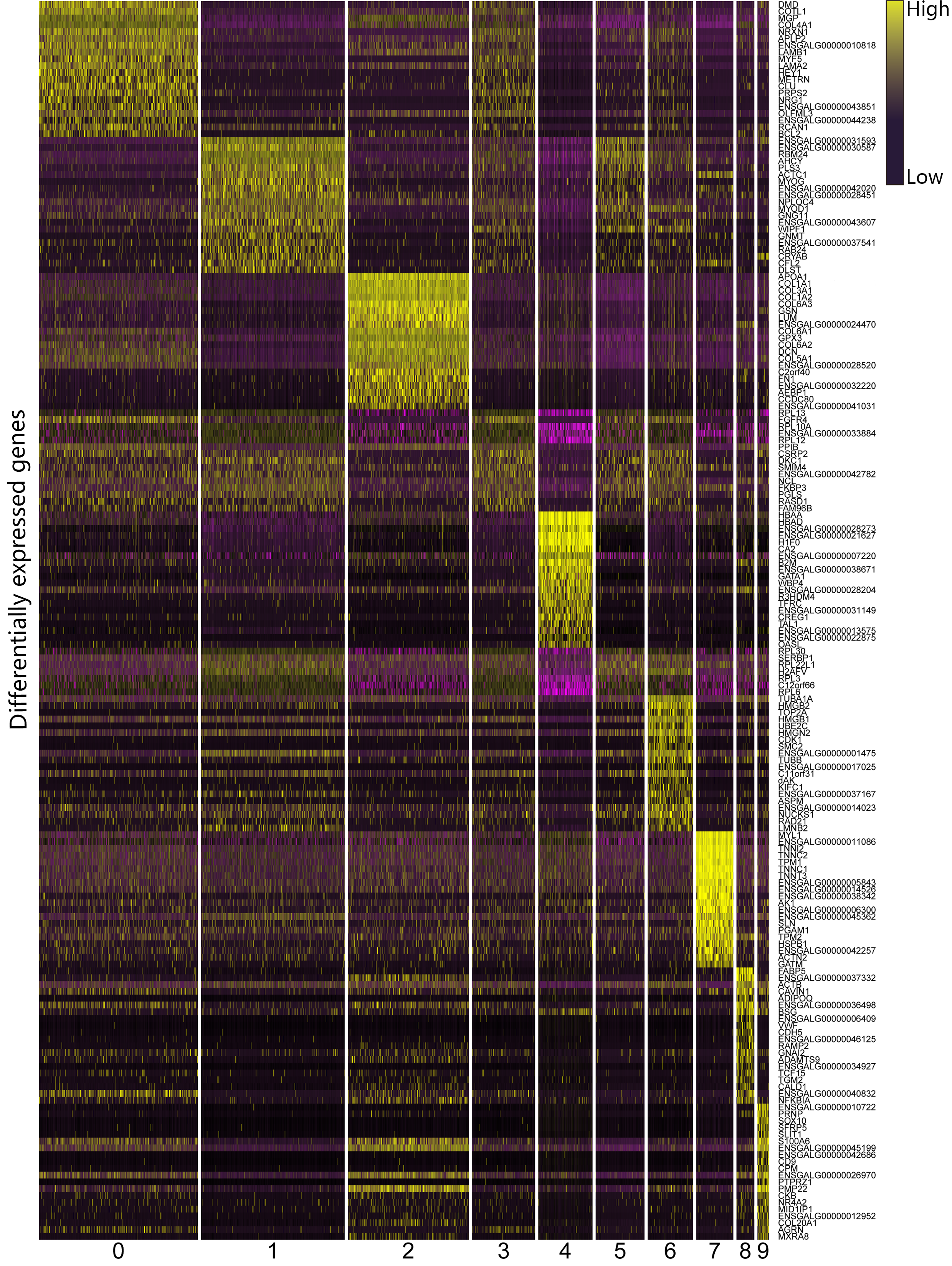

Supplement: Supplementary file 2 — Additional file 2: Figure S2. Heatmap of the top 20 up-regulated genes in each cluster at D5. The abscissa represents the cell clusters, and the ordinate represents the up-regulated genes in each cluster. The color changes from purple to yellow, indicating the gradual increase in gene expression. [file 12864_2020_7136_MOESM2_ESM.tif]

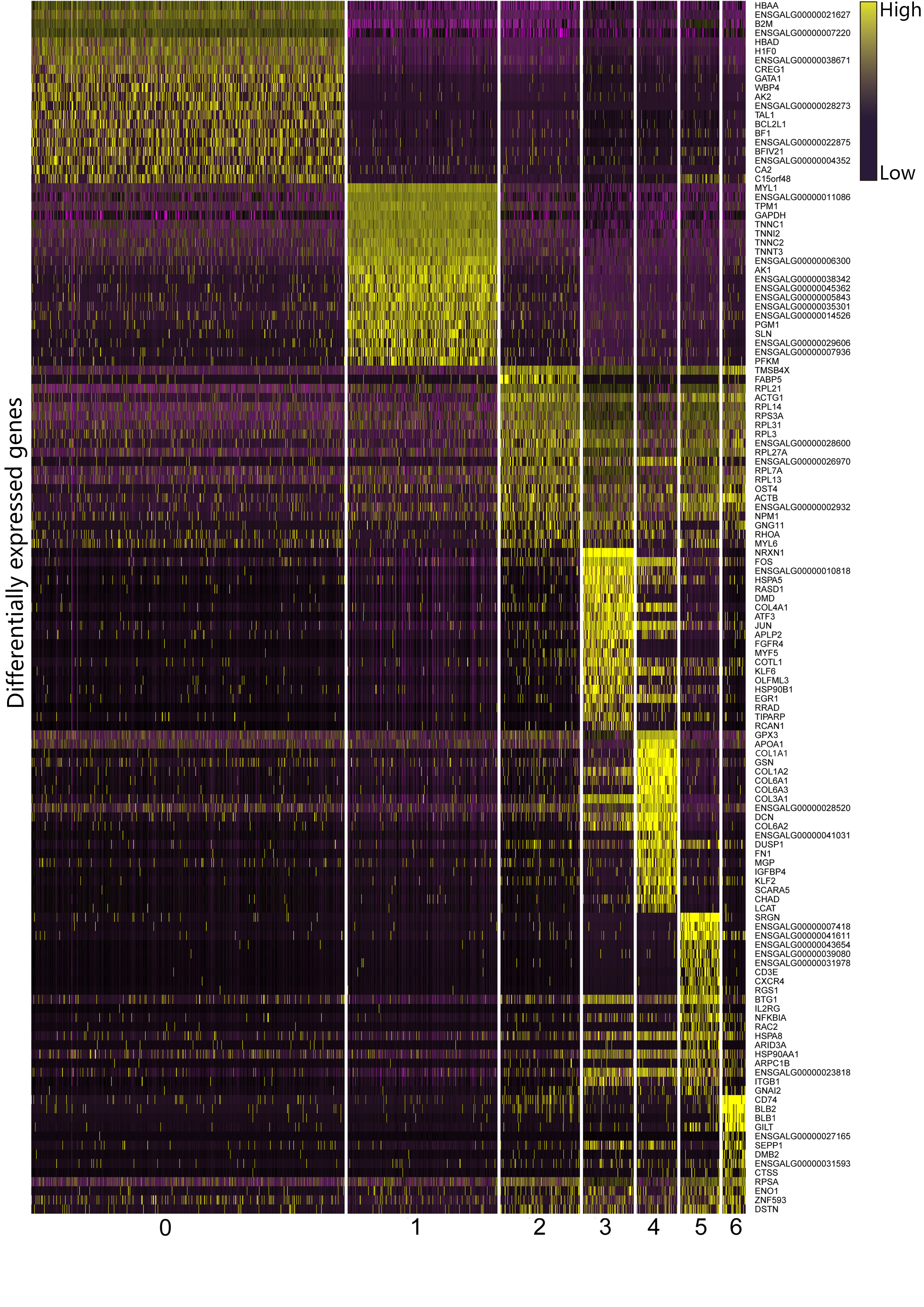

Supplement: Supplementary file 3 — Additional file 3: Figure S3. Heatmap of the top 20 up-regulated genes in each cluster at D100. The abscissa represents the cell clusters, and the ordinate represents the up-regulated genes in each cluster. The color changes from purple to yellow, indicating the gradual increase in gene expression. [file 12864_2020_7136_MOESM3_ESM.tif]

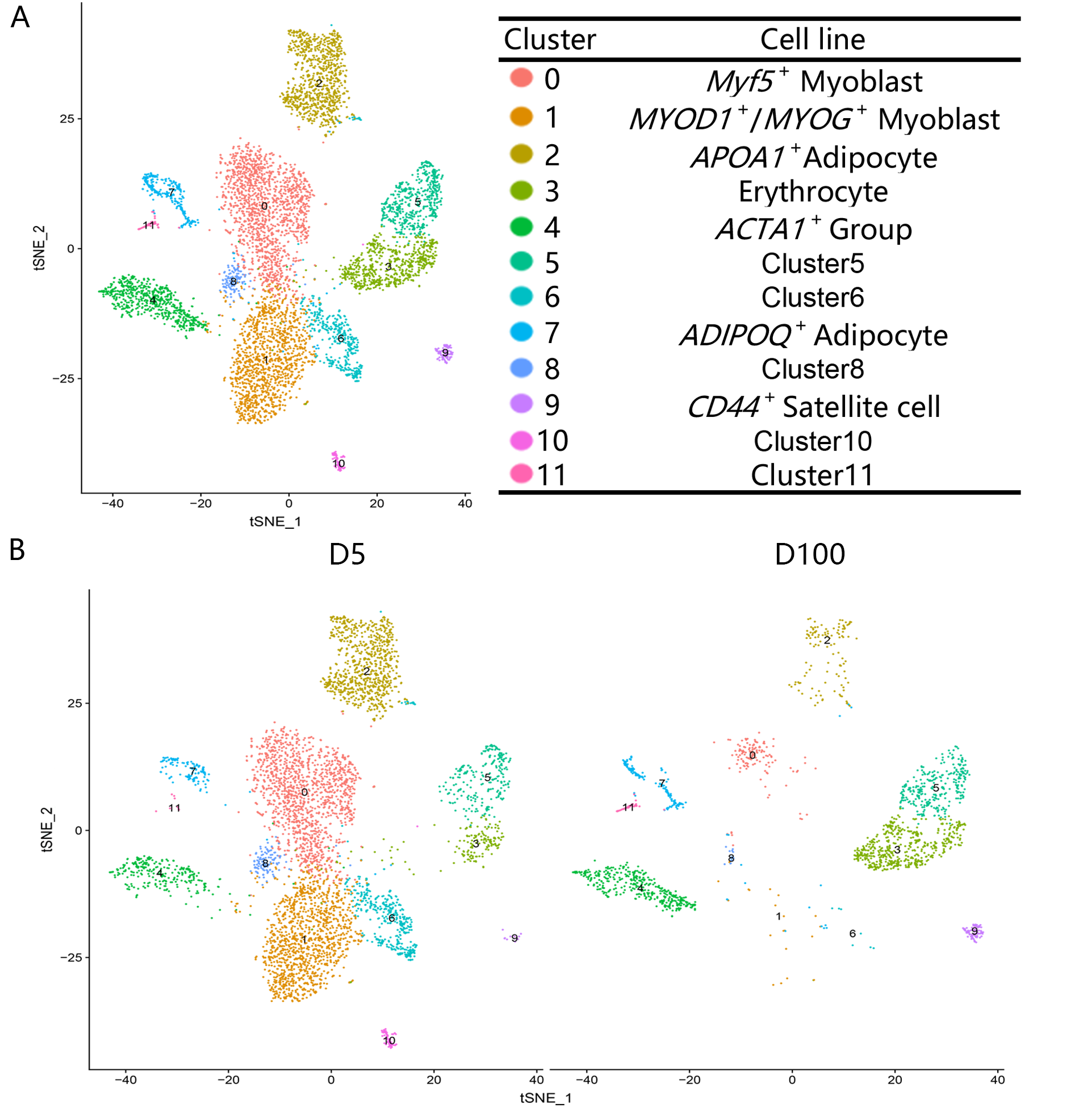

Supplement: Supplementary file 4 — Additional file 4: Figure S4. The t-SNE analysis of the data of two samples. Fig. A was the t-SNE result integrative results. Fig. B was the t-SNE results of D5 and D100, respectively. They merged to generate Fig. A. [file 12864_2020_7136_MOESM4_ESM.tif]

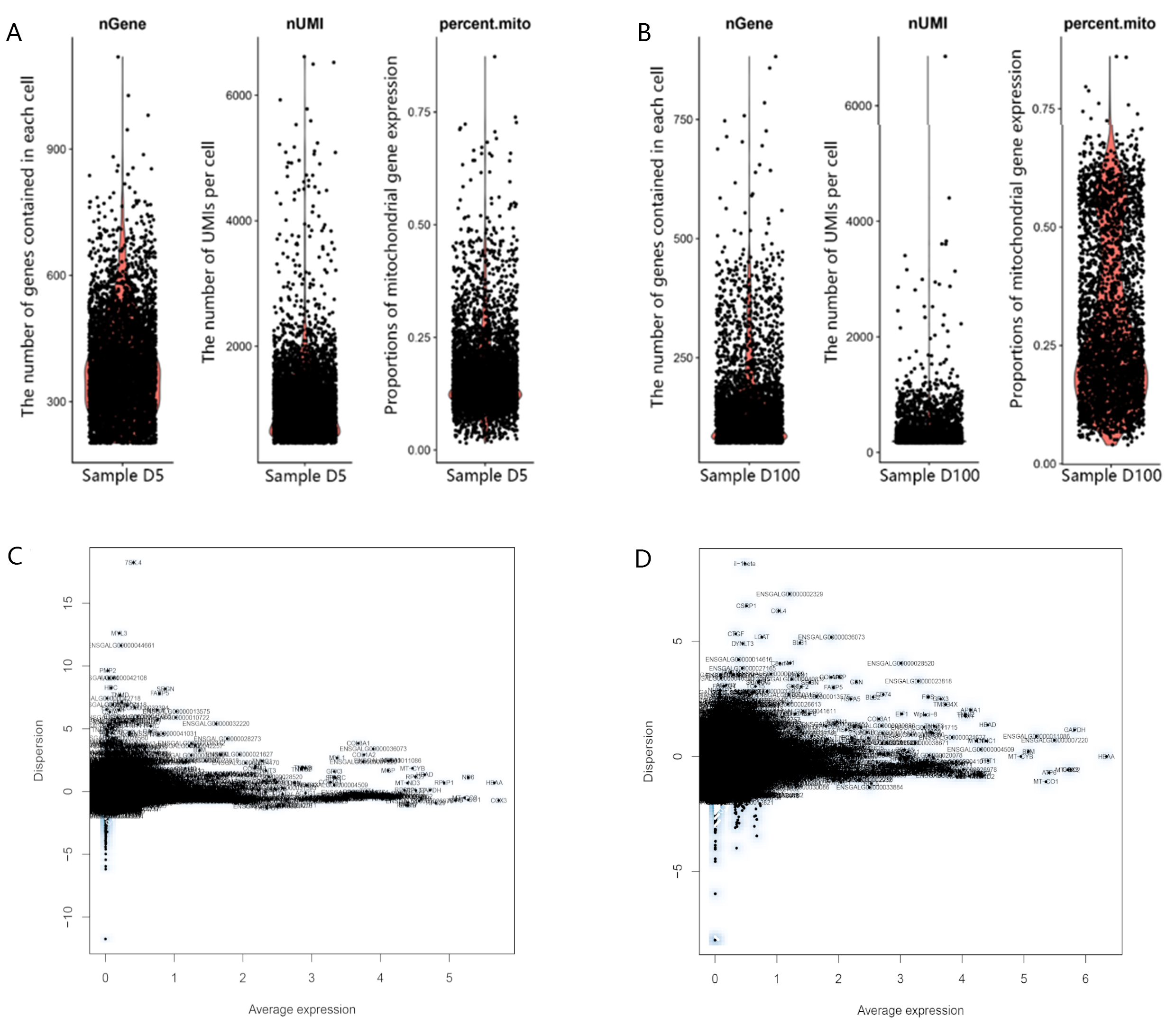

Supplement: Supplementary file 5 — Additional file 5: Figure S5. Distribution of data for quality assessment and data processing. A and B present the violin plots for the number of genes, UMI, and proportions of mitochondrial gene expression in detected cells at D5 and D100, respectively. C and D are the scatter plots of gene dispersion at D5 and D100, respectively. The ordinate represents the dispersion of the gene expression. [file 12864_2020_7136_MOESM5_ESM.tif]

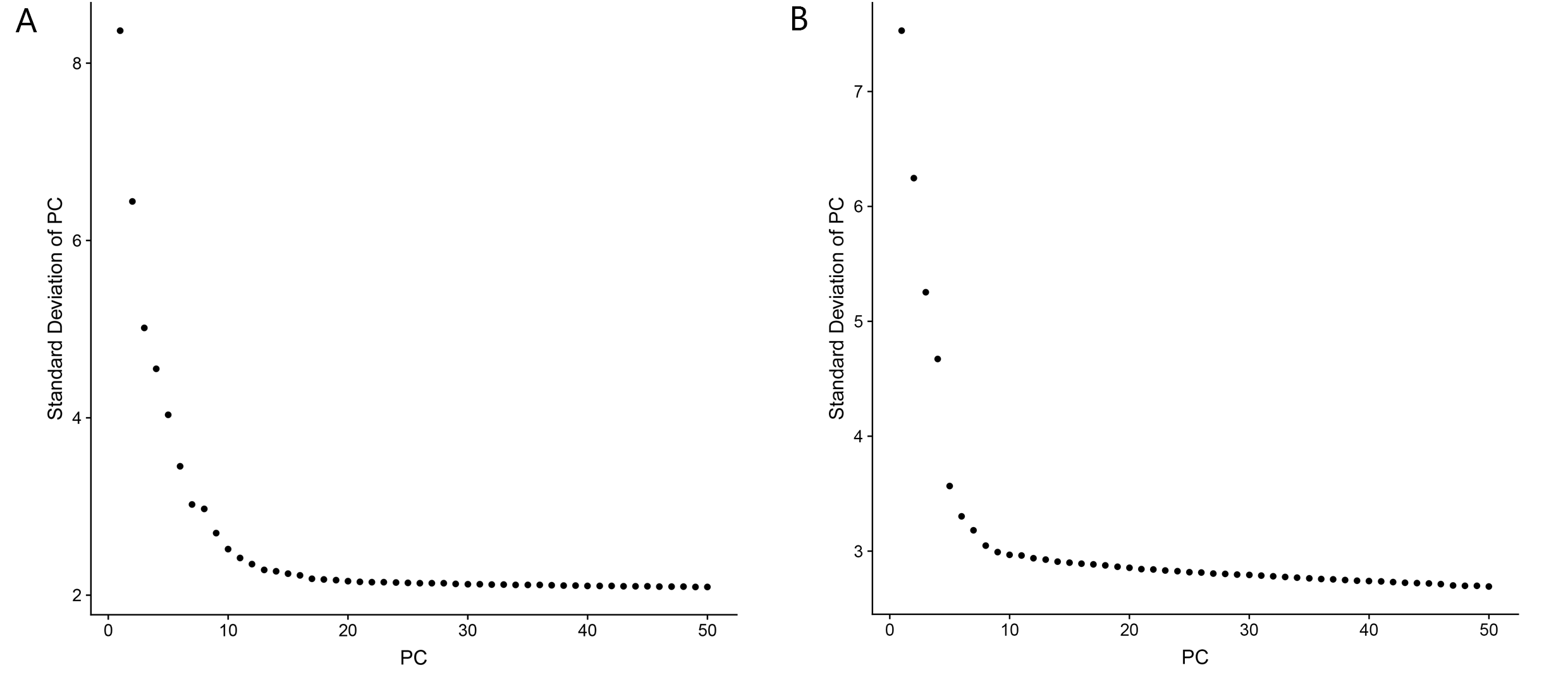

Supplement: Supplementary file 6 — Additional file 6: Figure S6. Principal component standard deviation scatter plot at D5 (Fig. A) and D100 (Fig. B). The abscissa represents the principal component, and the ordinate represents the standard deviation of different principal components. [file 12864_2020_7136_MOESM6_ESM.tif]
